# Clinical epidemiology and outcomes of patients with gastric intestinal metaplasia in the Los Angeles County System

**DOI:** 10.1186/s12876-023-02797-1

**Published:** 2023-05-19

**Authors:** Preeti Prakash, Shailavi Jain, Harry Trieu, Kenneth Chow, Deepthi Karunasiri, Tom Liang, Evan Yung, Holli Mason, Hongying Tan, James H. Tabibian

**Affiliations:** 1 David Geffen School of Medicine at the University of California Los Angeles (UCLA), Los Angeles, CA USA; 2 Department of Medicine, Massachusetts General Hospital, MA Boston, USA; 3 Keck School of Medicine at the University of Southern California, Los Angeles, CA USA; 4 Department of Medicine, Harbor-UCLA Medical Center, Torrance, CA USA; 5 Department of Pathology, Olive-View-UCLA Medical Center, Sylmar, CA USA; 6 Department of Pathology, Los Angeles County-University of Southern California Medical Center, Los Angeles, CA USA; 7 Department of Pathology, Harbor-UCLA Medical Center, Torrance, CA USA; 8 Division of Gastroenterology, Department of Medicine, Olive-View-UCLA Medical Center, Sylmar, CA USA

**Keywords:** Endoscopy, Gastric cancer, Surveillance, Risk Factors, Disparities, Healthcare

## Abstract

**Background:**

Gastric intestinal metaplasia (GIM) is a precursor to gastric adenocarcinoma (GAC). In the United States, there is no consensus on the utility of surveillance for GIM, and minority populations most affected by GAC are understudied. Our aims were to define clinical and endoscopic features, surveillance practices, and outcomes in patients with GIM in a multicenter safety-net system.

**Methods:**

We identified patients with biopsy-proven GIM between 2016–2020 at the three medical centers comprising Los Angeles County Department of Health Services. Demographics, findings at index esophagogastroduodenoscopy (EGD) first showing GIM, recommended interval for repeat EGD*,* and findings at repeat EGD were abstracted. Descriptive statistics were performed to characterize our cohort. T-tests and chi-squared (χ^2^) tests were used to compare patients with and without multifocal GIM.

**Results:**

There were 342 patients with newly-diagnosed biopsy-proven GIM, 18 (5.2%) of whom had GAC at index EGD. Hispanic patients comprised 71.8% of patients. For most patients (59%), repeat EGD was not recommended. If recommended, 2–3 years was the most common interval. During a median time to repeat EGD of 13 months and cumulative follow-up of 119 patient-years, 29.5% of patients underwent at least one repeat EGD, of whom 14% had multifocal GIM not previously detected. Progression to dysplasia or GAC was not detected in any patients.

**Conclusion:**

In a predominantly minority population with biopsy-proven GIM, there was a 5% incidence of GAC on index EGD. Though progression to neither dysplasia nor GAC was detected, there was significant variability in endoscopic sampling and surveillance practices.

**Supplementary Information:**

The online version contains supplementary material available at 10.1186/s12876-023-02797-1.

## Introduction

Gastric cancer is the fifth most common cancer and the third leadingcause of cancer-related deaths worldwide [1]. It occurs more frequently in men and people of African-American, Hispanic, and Asian backgrounds [2]. In the United States (US), the incidence of gastric cancer is approximately 26,000 cases per year, representing the 15th most common cancer type [1]. Gastric intestinal metaplasia (GIM) is a known precursor lesion to gastric adenocarcinoma (GAC). Esophagoduodenoscopy (EGD) provides the ability to detect this precursor lesion and obtain targeted biopsies. However, given the dearth of epidemiological and other clinical data, there is no consensus on neither the utility of GAC screening nor optimal surveillance intervals in the US for patients with biopsy-proven GIM [3, 4].

The American Society for Gastrointestinal Endoscopy (ASGE) has recommended surveillance EGD in patients with biopsy-proven GIM who have increased risk for GAC due to ethnic background or family history but does not specify optimal surveillance intervals [3]. Other international societies have recommended individualized screening and surveillance strategies for people with extensive GIM, incomplete GIM subtype, family history of GAC, or persistent *Helicobacter pylori (H. pylori)*gastritis, in addition to ethnic minorities and first-generation immigrants from countries with a high incidence of GAC [5, 6]. In light of the limited available data, the American Gastroenterological Association published guidelines recommending the eradication of *H. pylori*in patients with GIM but no definitive recommendations for endoscopic surveillance [4]. Though GAC is overall rare in the US compared to other cancers, minority populations which are most affected by it are relatively understudied [7–9]. Considering modern endoscopic capabilities, the presence of a sizeable minority population receiving healthcare in the US, and the high mortality rates associated with GAC, it is important to gain a better understanding of the clinical features and outcomes of GIM in order to inform optimal endoscopic surveillance strategies. This is particularly important in populations at increased risk for GIM and/or GAC, such as the Hispanic-predominant Los Angeles Department of Health Services (LADHS) patient population [10].

We performed a multicenter, retrospective cohort study of patients seen at the three medical centers comprising the LADHS system with biopsy-proven GIM. Our aim was to examine the demographics, clinical features, and outcomes of these patients, as this may ultimately inform the utility of endoscopic surveillance practices in minority ethnic populations.

## Methods

### Ethics approval

This study received approval from the Institutional Review Board of the Olive View-UCLA Education and Research Institute and was performed in accordance with the ethical standards of the 1964 Declaration of Helsinki and its amendments.

### Selection criteria

Patients over 18 years of age with biopsy-proven GIM diagnosed at Olive View-UCLA Medical Center (OVMC), Harbor-UCLA Medical Center (HUMC), and Los Angeles County + USC Medical Center (LAC + USC) between 2016–2020 were included in our study. Patients under 18 years of age, without a biopsy-proven diagnosis of GIM, and/or had a pathologic diagnosis made only at an institution outside of the LADHS system and unable to be confirmed through our records were excluded from the study.

### Study outcomes

The primary study outcome was progression of GIM to GAC at surveillance (repeat) EGD. Secondary outcomes included progression of GIM to low- or high-grade dysplasia at repeat EGD and progression of GIM to multifocal GIM at repeat EGD.

### Study variables and data abstraction

The pathology log repositories at OVMC, HUMC, and LAC + USC were queried for patients diagnosed with GIM between 2016 and 2020. The electronic medical records for each patient were then reviewed and the following demographic and clinical data were abstracted: age, sex, insurance status, race/ethnicity, body mass index, smoking history, alcohol use history, history of *H. pylori* infection and treatment, personal history of gastrointestinal (GI) or non- GI cancer, and family (first degree relative) history of gastric, non-gastric GI, and non-GI cancers.

We reviewed EGD reports from the first EGD with biopsies positive for GIM (index EGD) and collected the following data: age, date, presence of symptoms at the time of index EGD, indication for index EGD, location of endoscopic abnormalities, and type of endoscopic abnormalities. We subsequently reviewed pathology reports and abstracted the following variables: biopsy location, presence of low- or high- grade dysplasia, presence of multifocal GIM, presence of *H. pylori,* and presence of carcinoma. If mentioned in the EGD report or follow-up note, the recommended time interval for repeat EGD for GIM was recorded. Laboratory data at the time of index EGD, including hemoglobin at the time of index EGD was abstracted.

If a patient underwent repeat EGD, the corresponding endoscopy and pathology reports were reviewed and the following data were abstracted: date of repeat EGD, and endoscopic and histologic findings at repeat EGD including the development of low- or high-grade dysplasia, multifocal GIM, and/or carcinoma. Finally, for each patient, whether a final diagnosis of GAC was determined and date of last clinic encounter were collected.

### Data analysis

We performed descriptive statistics to characterize the study sample. T-tests and chi-squared $\left({x}^{2}\right)$ tests were used to compare demographic, clinical, laboratory, and endoscopic parameters in patients with and without progression of focal to multi-focal GIM at repeat EGD. Statistical analyses were performed using Stata/IC 16.1 (StataCorp, College Station, TX, United States).

## Results

### Sample characteristics

A total of 342 patients across all three LADHS medical centers with biopsy-proven GIM were included in the study. Male patients accounted for 51.5% of the study sample, and the median age at the time of index EGD was 59 years (Table 1). The majority of patients were Hispanic (71.8%), followed by Asian (12.4%), White (6.2%), Middle Eastern (4.7%), African American (2.9%), and other race (2.1%). One-third of patients had a documented history of *H. pylori* infection and 31.9% of patients had a history of gastroesophageal reflux disease (GERD). The majority of patients (61.9%) had no smoking history. The vast majority of patients (87.7%) had no personal history of cancer diagnosed prior to EGD. The most common indication for the index EGD was dyspepsia or gastroesophageal reflux symptoms (27.1%) followed by abdominal pain (25.7%) and iron deficiency anemia (23.4%).

**Table 1:** Characteristics of patients with biopsy-proven gastric intestinal metaplasia (*n* = 342)

**Male sex, n (%)**	176 (51.5%)
**Age at the time of index EGD, median (IQR)**	59 (52–65)
**Race, n (%)**	
White	21 (6.2%)
Hispanic	244 (71.8%)
Asian	42 (12.4%)
African American	10 (2.9%)
Middle Eastern/North African	16 (4.7%)
Other	7 (2.1%)
**Smoking history, n (%)**	
Never	212 (61.9%)
Current	44 (12.9%)
Former	85 (24.8%)
**History of GERD**	109 (31.9%)
**History of *H. Pylori*, n (%)**	
Yes	114 (33.3%)
No	228 (66%)
**Personal history of cancer diagnosed prior to endoscopy, n (%)**^a^	
No cancer	299 (87.7%)
Non-gastric GI cancer	14 (4.1%)
Non-GI cancer	29 (8.5%)
**Family history of 1st degree relative with cancer**	
No cancer	275 (80.7%)
Gastric cancer	15 (4.4%)
Non-gastric GI cancer	18 (5.3%)
Other cancer	38 (11.1%)
**Hemoglobin at the time of index EGD, median (IQR)**	12.8 (11.0–13.9)

^a^One patient had a history of both GI and non-GI cancers. One patient had a family history of gastric cancer and other cancer(s)

### Findings at index EGD

Of the 342 patients, 108 patients (31.6%) had gastric biopsies taken from more than one location during index EGD, and the remainder had biopsies taken from only one location (Table 2). GAC was present in 18 patients (5.3%), high-grade dysplasia in no patients, low-grade dysplasia in one patient (0.3%), and multifocal GIM in 53 patients (15.5%)*. H. pylori* infection was diagnosed on biopsy in 97 patients (28.4%). Repeat EGD was recommended for 41% of patients. The most common recommended time interval for repeat EGD was 2–3 years (24.2%), followed by one year (9.6%), 3–6 months (3.5%), 1–3 months (2.6%), and 5 years (0.5%).

**Table 2:** Findings at index EGD with biopsy-proven GIM and outcomes post-index EGD

**Indication for index EGD (Top 5)**	
Dyspepsia or gastroesophageal reflux, n (%)	93 (27.1%)
Abdominal pain, n (%)	88 (25.7%)
Iron deficiency anemia, n (%)	80 (23.4%)
Gastrointestinal bleeding, n (%)	52 (15.2%)
Weight loss, n (%)	36 (10.5%)
**Findings at index EGD**	
Low-grade dysplasia at index EGD, n (%)^a^	1 (0.3%)
High-grade dysplasia at index EGD, n (%)	0 (0.0%)
Multifocal GIM at index EGD, n (%)^b^	53 (15.5%)
GAC at index EGD, n (%)	18 (5.3%)
*H. pylori* on biopsy, n (%)	97 (28.4%)
**Recommended interval for repeat EGD for GIM surveillance**	
EGD not recommended	202 (59%)
1–3 months	9 (2.6%)
3–6 months	12 (3.5%)
1 year	33 (9.6%)
2–3 years	83 (24.2%)
5 years	2 (0.5%)
**Patients with repeat EGD with biopsies, n (%)**	81 (23.6%)
**Months from index to repeat EGD, median (IQR)**	13 (3–29)
**Months from index EGD to last clinic follow-up, median (IQR)**	14.7 (2.2–42.7)
**Findings at repeat EGD**	
Low-grade dysplasia at repeat EGD, n (%)	0 (0)
High-grade dysplasia at repeat EGD, n (%)	0 (0)
Multifocal GIM at repeat EGD not found at index EGD, n (%)^c^	10 (14%)
GAC at repeat EGD not found at index EGD, n (%)	0 (0)

^a^One patient who had low-grade dysplasia at index EGD underwent repeat EGD but did not have biopsies obtained at that time

^b^There were 108 patients (31.6%) who had multifocal biopsies taken at index EGD

^c^Of these 10 patients, 3 patients had multifocal biopsies at index EGD

### Outcomes at repeat EGD

Excluding the patients with a diagnosis of GAC at index EGD, 81 patients (23.6%) underwent repeat EGD with biopsies (Table 2). The median time from index to repeat EGD was 13 months (interquartile range 3–29 months). Cumulative follow-up for these patients was 119.3 patient-years. At repeat EGD, no patients were found to have low- or high-grade dysplasia nor GAC. Multifocal GIM was found in 18 patients at repeat EGD. Of these 18 patients, 10 patients had multifocal GIM at repeat EGD that was not previously diagnosed at index EGD. However, only three of these 10 patients had biopsies taken in more than one location at their index EGD.

### Characteristics of patients with and without multifocal GIM at repeat EGD

Among patients with multifocal GIM at repeat EGD, 33.3% were male, whereas among those without multifocal GIM at repeat EGD, 47.6% were male (Table 3). The majority of patients with or without multifocal GIM at repeat EGD had no prior smoking history and no family history of cancer. Half of patients with multifocal GIM at repeat EGD had a history of *H. pylori* infection compared to 31.7% of patients who did not have multifocal GIM at repeat EGD. In patients with multifocal GIM at repeat EGD, 50% of patients were recommended to undergo repeat EGD in 2–3 years, 16.7% in one year, and 22.2% had no recommendation for repeat EGD at time of index EGD. In patients without multifocal GIM at repeat EGD, 30.2% were recommended to undergo repeat EGD in 2–3 years, 22.2% in one year, and 36.5% of patients had no recommendation for repeat EGD at the time of index EGD.

**Table 3:** Comparison of patients with and without multifocal GIM

	**With multifocal GIM at repeat EGD (*n* = 18)**	**Without multifocal GIM at repeat EGD (*n* = 63)**
**Male, n (%)**	6 (33.3%)	30 (47.6%)
**Age at index EGD, median (IQR)**	58 (55 – 68)	58 (53 – 64)
**Smoking history, n (%)**		
Never	14 (77.8%)	41 (65.1%)
Current	1 (5.6%)	10 (15.9%)
Former	3 (16.7%)	12 (19.0%)
**History of GERD**	7 (38.9%)	23 (36.5%)
**History of *H. pylori* infection**	9 (50.0%)	20 (31.7%)
**Family history of 1**^**st**^** degree relative with cancer**		
No family history	14 (77.8%)	46 (74.2%)
Gastric cancer	0 (0.0%)	3 (4.8%)
Non-gastric GI cancer	1 (5.6%)	6 (9.7%)
Other cancer	3 (16.7%)	8 (12.9%)
**Hemoglobin at time of index EGD, median (IQR)**	12.7 (9.7 – 13.4)	12.5 (11.2 – 13.8)
**Recommended interval for repeat EGD for GIM surveillance**		
No recommendation	4 (22.2%)	23 (36.5%)
< 3 months	0 (0.0%)	3 (4.8%)
3–6 months	1 (5.6%)	5 (7.9%)
1 year	3 (16.7%)	14 (22.2%)
2–3 years	9 (50.0%)	19 (30.2%)
5 years	1 (5.6%)	0 (0.0%)
**Location of endoscopic abnormality observed at index EGD**		
No abnormalities	2 (11.1%)	6 (9.8%)
Antrum	5 (27.8%)	28 (45.9%)
Body	6 (33.3%)	19 (31.1%)
Pylorus	2 (11.1%)	4 (6.6%)
Cardia	3 (16.7%)	4 (6.6%)
Fundus	4 (22.2%)	5 (8.2%)
Generalized	5 (27.8%)	13 (21.3%)
Angularis	0 (0.0%)	4 (6.6%)
**Type of endoscopic abnormality observed at index EGD**		
No abnormalities	2 (11.1%)	6 (10.0%)
Erythema	5 (27.8%)	6 (10.0%)
Nodularity	0 (0.0%)	11 (18.3%)
Erythema and erosions	3 (16.7%)	12 (20.0%)
Atrophic/decreased folds	3 (16.7%)	8 (13.3%)
Ulcer	3 (16.7%)	12 (20.0%)
Polyps	1 (5.6%)	9 (15.0%)
Other^a^	4 (22.2%)	13 (21.6%)

^a^Other included erythema and nodularity, gastropathy unspecified, thickened folds, or mottled appearance

### Endoscopic findings of patients with and without multifocal GIM at repeat EGD

The most common location of endoscopic abnormality at index EGD in patients with multifocal GIM at repeat EGD was the body (33.3%), generalized (27.8%), and the antrum (27.8%). The most common location of endoscopic abnormality at index EGD in patients *without* multifocal GIM at repeat evaluation was the antrum (45.9%), body (31.1%), and generalized (21.3%).

In patients with multifocal GIM diagnosed at repeat EGD, the most common endoscopic abnormality seen at index EGD was erythema (27.8%), followed by erythema and erosions, decreased folds, and ulceration, all seen in 16.7% of patients, respectively. In patients *without* multifocal GIM diagnosed at repeat EGD, the most common abnormality seen at index EGD was erythema and erosions (20%), ulceration (20%), and nodularity (18.3%).

### Characteristics and endoscopic findings of patients diagnosed with GAC on index EGD

Of the 18 patients who were diagnosed with GAC on index EGD, 61.1% were male and median age was 71 years (Table 4). Approximately half of these patients (55.6%) had either a current or former history of smoking. Most patients (66.7%) had no family history of a first degree relative with cancer. No patients had a first degree relative with gastric cancer, but 27.8% of patients had a family history of a first degree relative with non-gastric GI cancer. Median hemoglobin at the time of index EGD was 12.7, which was essentially equivalent to the median hemoglobin of all patients diagnosed with GIM on index EGD. Abdominal pain was the most common indication for index EGD amongst patients diagnosed with GAC, followed by weight loss or constitutional symptoms. The most common location with the positive biopsy was the antrum, followed by the fundus.

**Table 4:** Characteristics and endoscopic findings of patients diagnosed with gastric adenocarcinoma at index EGD

	**With gastric adenocarcinoma on index EGD (*n* = 18)**
**Male, n (%)**	11 (61.1%)
**Age at index EGD, median (IQR)**	71 (64 – 74)
**Smoking history, n (%)**	
Never	8 (44.4%)
Current	3 (16.7%)
Former	7 (38.9%)
**History of GERD**	7 (38.9%)
**History of *H. pylori* infection**	1 (5.5%)
**Family history of 1**^**st**^** degree relative with cancer**	
No family history	12 (66.7%)
Gastric cancer	0 (0.0%)
Non-gastric GI cancer	5 (27.8%)
Other cancer	1 (5.5%)
**Hemoglobin at time of index EGD, median (IQR)**	12.7 (10.9 – 13.9)
**Indication for index EGD (Top 5)**	
Abdominal pain	8 (44.4%)
Weight loss or constitutional symptoms	7 (38.9%)
Abnormality on non-invasive imaging	6 (33.3%)
Early satiety or bloating	5 (27.8%)
GI bleeding	3 (16.7%)
Iron deficiency anemia	3 (16.7%)
**Location of endoscopic abnormality observed at index EGD**	
No abnormalities	0 (0.0%)
Antrum	9 (50.0%)
Body	3 (16.7%)
Pylorus	1 (5.5%)
Cardia	1 (5.5%)
Fundus	4 (22.2%)
Generalized	1 (5.5%)
Angularis	0 (0.0%)
**Type of endoscopic abnormality observed at index EGD**	
Erythema	2 (11.1%)
Nodularity	5 (27.8%)
Erythema and erosions	0 (0.0%)
Atrophic/decreased folds	3 (16.7%)
Gastropathy, not otherwise specified	3 (16.7%)
Ulcer	3 (16.7%)
Thickened folds	1 (5.5%)
Mottled appearance	4 (22.2%)
Other^a^	6 (33.3%)

^a^Other included visible tumor, friability, plaques, and pyloric stricture

## Discussion

In this multicenter retrospective cohort study of patients with biopsy-proven GIM, the majority of our cohort was comprised of Hispanic and Asian patients, two ethnic minorities known to have an increased risk of GAC [2]. In addition, a greater than average incidence of prior *H. pylori*infection was seen in our cohort, which is known to occur more frequently in ethnic minorities and is a recognized driver of GIM [11]. Our cohort also had a high incidence (5%) of GAC at the time of index EGD with biopsy-proven GIM, which corroborates the need to explore the utility of GIM surveillance particularly in ethnic minorities [12]. The recommended interval for repeat EGD for GIM surveillance varied greatly in our study population, with no recommendation for further GIM surveillance in a majority of patients, followed by 2–3 years as the most common time interval if surveillance was recommended. This variability in surveillance recommendations is likely driven by the lack of consensus in the US on the need for GIM surveillance and the optimal time intervals for surveillance when pursued.

Of the patients who underwent a repeat EGD with biopsy during the study period, none developed low- or high-grade dysplasia nor GAC. A small proportion (14%) had multifocal GIM detected at repeat EGD, though it is possible a portion of these patients already had multifocal GIM at index EGD that was not appreciated (e.g. due to limited biopsies). Our data suggest that the progression of GIM to low- or high-grade dysplasia and GAC may not occur within a 1–2 year period, and the same likely applies to progression to multifocal GIM. However, we are limited in our ability to propose an optimal surveillance interval for GIM given that only a minority of patients diagnosed with GIM underwent surveillance EGD as well as the relatively limited median follow-up time of patients in our study.

In our subgroup analysis of patients with multifocal GIM found at repeat EGD, in comparison to patients without multifocal GIM at repeat EGD, there was a greater percentage of females with multifocal GIM, which is not an established risk factor for GIM or GAC. Similar to prior literature, there was a greater percentage of patients with a history of *H. pylori*amongst those who were diagnosed with multifocal GIM at repeat EGD [1, 3]. Smoking history and a family history of GAC, identified as risk factors for GAC and GIM progression in prior studies, were not reproduced as significantly more prevalent in patients with multifocal GIM at repeat EGD [13, 14].

It should be emphasized that most patients in our study had gastric biopsies taken in one location at index EGD. Of the patients who *did*have multifocal GIM at repeat EGD, the majority did not have biopsies taken in more than one location at index EGD. Moreover, there were patients without visualized abnormalities endoscopically who had multifocal GIM diagnosed on biopsy. This highlights the variability in the number of biopsies routinely obtained during diagnostic EGD when a diagnosis of GIM is not known [15, 16]. It also suggests that the true incidence of multifocal GIM may be higher than what is diagnosed based on current biopsy practices.

Our study had several limitations. Although we abstracted data from a five-year time period and achieved a cumulative follow-up of 119 patient-years, the median duration of follow-up from the time of index EGD to repeat EGD and last clinic encounter was less than two years. Additionally, the limited number of follow-up EGDs and few patients with documented progression to multifocal GIM precluded the use of multiple logistic regression to model risk factors for progression and time to progression of GIM that could better inform surveillance intervals in our population of interest. Furthermore, univariate analyses to compare characteristics between progressors and non-progressors were not meaningful given that the repeated use of hypothesis testing increases the chance of a Type I error and is not a suitable alternative when the number of outcomes is insufficient for multivariate analysis (Supplementary Table 1). These limitations highlight the challenges in abstracting robust data in safety-net settings where low follow-up rates are common and the importance of developing strategies to mitigate this [17]. Furthermore, cost was not measured or modeled in our study; however, we believe that appropriately spacing out surveillance EGDs based on the natural history of GIM would not only provide healthcare cost benefits, but also decrease burden on patients.

## Conclusions

In summary, we describe a multicenter, retrospective study of patients with biopsy-proven GIM in a large, underserved county health system with predominantly minority patients, with a historically higher incidence of GAC. Our cohort demonstrated a 5% incidence of GAC at the time of index EGD with biopsy-proven GIM, emphasizing a need for more informed surveillance guidelines applicable to this patient population. During our study period and a cumulative follow-up of 119 patient-years, there was no apparent progression to low or high-grade dysplasia nor GAC and low incidence of multifocal GIM. Our data suggest that progression to dysplasia or GAC is unlikely to occur within a 1–2 year period. However, limitations in our dataset preclude identification of optimal surveillance intervals. Our study highlights variability in surveillance recommendations and biopsy sampling at index EGD. The limited median follow-up periods in our study also emphasizes the challenges in collecting longitudinal data in safety-net settings that has likely contributed to the paucity of data in ethno-racial and other minority populations that could otherwise inform evidence-based surveillance practices. Randomized controlled trials incorporating a diverse patient population may be needed in order to better elucidate the need for, approach to, and frequency of surveillance endoscopy for biopsy-proven GIM.

## Supplementary Information

**Additional file 1:** **Supplementary Table 1.** Univariate analysis of patients with andwithout multifocal GIM or carcinoma at repeat EGD.

## Data Availability

The datasets used and/or analysed during the current study are available from the corresponding author on reasonable request.

## Abbreviations

ASGE: American Society for Gastrointestinal Endoscopy
EGD: Esophagoduodenoscopy
GERD: Gastroesophageal reflux disease
GIM: Gastric intestinal metaplasia
H. pylori: Helicobacter pylori
HUMC: Medical Center Harbor-UCLA Medical Center
Index EGD: First EGD with biopsies positive for GIM
LAC + USC: Los Angeles County + USC Medical Center
LADHS: Los Angeles County Department of Health Services
OVMC: Olive View-UCLA
US: United States
