# Supplementary material for: Clinical epidemiology and outcomes of patients with gastric intestinal metaplasia in the Los Angeles County System
Source: BMC Gastroenterol. 2023 May 19;23:165. doi: 10.1186/s12876-023-02797-1 (PMC10197307; doi:10.1186/s12876-023-02797-1)
Supplement: Supplementary file 1 — Additional file 1: Supplementary Table 1. Univariate analysis of patients with andwithout multifocal GIM or carcinoma at repeat EGD. [file 12876_2023_2797_MOESM1_ESM.docx]

**Supplementary table 1.** Univariate analysis of patients with and without multifocal GIM or carcinoma at repeat EGD

|  | **Without multifocal GIM or carcinoma at repeat EGD (n=63)** | **Multifocal GIM without carcinoma at repeat EGD (n=18)** | **p-value** |
| --- | --- | --- | --- |
| **Male, n (%)** | 30 (47.6%) | 6 (33.3%) | 0.282 |
| **Age at index EGD, median (IQR)** | 58 (53 – 64) | 58 (55 – 68) | 0.445 |
| **Smoking history, n (%)** |  |  | 0.479 |
| Never | 41 (65.1%) | 14 (77.8%) |  |
| Current | 10 (15.9%) | 1 (5.6%) |  |
| Former | 12 (19.0%) | 3 (16.7%) |  |
| **History of GERD** | 23 (36.5%) | 7 (38.9%) | 0.854 |
| **History of *H. pylori* infection** | 20 (31.7%) | 9 (50.0%) | 0.154 |
| **Family history of 1^st^ degree relative with cancer** |  |  |  |
| No family history | 46 (74.2%) | 14 (77.8%) | 0.757 |
| Gastric cancer | 3 (4.8%) | 0 (0.0%) | 0.341 |
| Non-gastric GI cancer | 6 (9.7%) | 1 (5.6%) | 0.722 |
| Other cancer | 8 (12.9%) | 3 (16.7%) | 0.683 |
| **Hemoglobin at time of index EGD, median (IQR)** | 12.5 (11.2 – 13.8) | 12.7 (9.7 – 13.4) | 0.343 |
| **Recommended interval for repeat EGD for GIM surveillance** |  |  |  |
| No recommendation | 23 (36.5%) | 4 (22.2%) | 0.257 |
| <3 months | 3 (4.8%) | 0 (0.0%) | 0.345 |
| 3-6 months | 5 (7.9%) | 1 (5.6%) | 0.734 |
| 1 year | 14 (22.2%) | 3 (16.7%) | 0.610 |
| 2-3 years | 19 (30.2%) | 9 (50.0%) | 0.119 |
| 5 years | 0 (0.0%) | 1 (5.6%) | 0.060 |
| **Location of endoscopic abnormality observed at index EGD** |  |  |  |
| No abnormalities | 6 (9.8%) | 2 (11.1%) | 0.875 |
| Antrum | 28 (45.9%) | 5 (27.8%) | 0.171 |
| Body | 19 (31.1%) | 6 (33.3%) | 0.861 |
| Pylorus | 4 (6.6%) | 2 (11.1%) | 0.522 |
| Cardia | 4 (6.6%) | 3 (16.7%) | 0.185 |
| Fundus | 5 (8.2%) | 4 (22.2%) | 0.100 |
| Generalized | 13 (21.3%) | 5 (27.8%) | 0.565 |
| Incisura angularis | 4 (6.6%) | 0 (0.0%) | 0.265 |
| **Type of endoscopic abnormality observed at index EGD** |  |  |  |
| No abnormalities | 6 (10.0%) | 2 (11.1%) | 0.892 |
| Erythema | 6 (10.0%) | 5 (27.8%) | 0.057 |
| Nodularity | 11 (18.3%) | 0 (0.0%) | 0.050* |
| Erythema and erosions | 12 (20.0%) | 3 (16.7%) | 0.753 |
| Atrophic/decreased folds | 8 (13.3%) | 3 (16.7%) | 0.722 |
| Ulcer | 12 (20.0%) | 3 (16.7%) | 0.753 |
| Polyps | 9 (15.0%) | 1 (5.6%) | 0.293 |
| Other* | 4 (22.2%) | 13 (21.6%) |  |

*Other included erythema and nodularity, gastropathy unspecified, thickened folds, or mottled appearance

Analyses were limited due to low number of repeat EGDs and a low number of patients with documented multifocal GIM at repeat EGD. Nodularity at index EGD was more common at index EGD in patients without multifocal GIM at repeat EGD (p-value 0.05).
